# Dominance of Objects over Context in a Mediotemporal Lobe Model of Schizophrenia

**DOI:** 10.1371/journal.pone.0006505

**Published:** 2009-08-04

**Authors:** Lucia M. Talamini, Martijn Meeter

**Affiliations:** 1 Department of Psychology, University of Amsterdam, Amsterdam, The Netherlands; 2 Department of Cognitive Psychology, Vrije Universiteit Amsterdam, Amsterdam, The Netherlands; University of Groningen, Netherlands

## Abstract

**Background:**

A large body of evidence suggests impaired context processing in schizophrenia. Here we propose that this impairment arises from defective integration of mediotemporal ‘what’ and ‘where’ routes, carrying object and spatial information to the hippocampus.

**Methodology and Findings:**

We have previously shown, in a mediotemporal lobe (MTL) model, that the abnormal connectivity between MTL regions observed in schizophrenia can explain the episodic memory deficits associated with the disorder. Here we show that the same neuropathology leads to several context processing deficits observed in patients with schizophrenia: 1) failure to choose subordinate stimuli over dominant ones when the former fit the context, 2) decreased contextual constraints in memory retrieval, as reflected in increased false alarm rates and 3) impaired retrieval of contextual information in source monitoring. Model analyses show that these deficits occur because the ‘schizophrenic MTL’ forms fragmented episodic representations, in which objects are overrepresented at the expense of spatial contextual information.

**Conclusions and Significance:**

These findings highlight the importance of MTL neuropathology in schizophrenia, demonstrating that it may underlie a broad spectrum of deficits, including context processing and memory impairments. It is argued that these processing deficits may contribute to central schizophrenia symptoms such as contextually inappropriate behavior, associative abnormalities, conversational drift, concreteness and delusions.

## Introduction

Schizophrenia is characterized by a complex symptomatology and mild to severe deficits in various domains of cognition. It has been suggested that at least some aspects of this pathology may be related to an underlying difficulty in processing context information [1]–[4]. Context here denotes all information that is spatially or temporally discontinuous with a given stimulus, but that may still contribute to its processing. In experimental situations, context can be defined concretely as all stimuli in a scene other than the target stimulus, including the room, background noise, objects or people, but also internal states of the subject.

Deficient context processing in schizophrenia is suggested by several clinical and experimental observations. For instance, behavior, thought and affect in schizophrenic patients may appear contextually inappropriate, particularly in delusional patients. Furthermore, thought-disordered patients display associational abnormalities and stray from the context in discourse (tangentiality). Since these phenomena can occur in patients in the absence of hallucinations, they are not secondary to altered perception.

Controlled studies indicate a diminished dependency of patient's responses on contextual information in various paradigms. For instance, in tasks in which semantically ambiguous words are presented in a context priming the less frequent meaning, schizophrenic patients display an abnormally strong tendency to erroneously select the more common meaning [2], [5]–[8]. Impairment is furthermore found on modified versions of the continuous performance test (AX-CPT) and Stroop task [2], [9]. In the first test, the task is to respond to a target (stimulus X) only if it is preceded by a specific cue (stimulus A). Patients make more errors in which they respond to the target if a different cue (stimulus B) is given, suggesting that they disregard the context supplied by the conditional cue. In the modified Stroop task, task instructions determine whether the subject should read the word or name the ink color. Again, patients with schizophrenia are less responsive to the context provided by task instructions than healthy participants and tend to select the more dominant, automatic response, namely to read the word. Finally, the absence of latent inhibition in patients with schizophrenia [10] has also been taken to reflect a contextual information processing deficit [4].

A certain disregard of context is also apparent in the domain of episodic memory. First, patients with schizophrenia display increased false alarm rates (recognition of stimuli that were not presented in the learning context), which suggest that context information imposes a weaker than normal constraint on retrieval [11], [12]. Second, several studies report impaired retrieval in schizophrenic patients for contextual aspects of events, including spatial, temporal [13]–[16] and “source” information [17]–[22].

The neural underpinnings of these dysfunctions have, thus far, remained obscure. In fact, it is not even clear whether the findings reflect the same [2] or dissociable deficiencies [9]. However, one common denominator of all mentioned tasks is that appropriate responses are based on configurations of complex stimuli that are spread out over time and space (this also holds for many real-life situations). Binding of such spatiotemporal configurations of stimuli is thought to depend on the hippocampus and surrounding regions [23]; brain areas that are also central to episodic memory [24], [25]. Neuropathology in these brain regions is well documented in schizophrenia [26] and has been related to episodic memory deficits [27], [28]. It seems possible that this neuropathology may also underlie context processing deficits.

In line with this notion, several studies suggest that episodic memory impairment in schizophrenia is largely due to abnormal encoding of episodes, even though retrieval may not be entirely spared [29]–[35]. This implies abnormalities in the neural representation of episodes and, thus, in the perception of events.

We have shown previously, using a computational model of the mediotemporal lobe (MTL), how neuropathological changes observed in schizophrenia, lead to a deficit in binding different aspects of an episode into one representation [28]. The MTL contains largely segregated pathways processing the objects and spatial configurations making up a scene. These pathways culminate in the perirhinal cortex (objects) and the parahippocampal cortex (spatial configurations), respectively [36], [37]. In most experimental paradigms, information carried by the second pathway is contextual in nature, but, nevertheless, contributes to task performance. The two pathways finally converge on the entorhinal cortex and hippocampus [38]–[40], where the information is bound into one episodic representation. There is evidence that in schizophrenia, the connections that underlie this convergence are severely reduced in number [26], whereas other connections within the MTL are relatively spared.

Implementation of these wiring abnormalities in a computational model of the medial temporal lobe, but not control manipulations, led to a memory deficit profile that closely resembles the one observed in patients with schizophrenia [28], with a moderate to severe deficit in free recall, a mild deficit in recognition and no preferential deficits in proactive interference [29]. Here, we investigate whether the same MTL neuropathology may also lead to schizophrenia-like deficits in context processing. Specifically, we investigate the hypothesis that abnormal wiring in the MTL results in a dominance of object information, at the expense of spatial configural information. This in turn, we will argue, leads to a weakening of contextual constraints on information processing.

As in our previous study, MTL neuropathology in schizophrenia is implemented in our MTL model as a 50% reduction of the convergent projections carrying object and spatial information from parahippocampal areas to the hippocampus. We investigate first how these wiring abnormalities affect the representation of episodic information at the network level. Subsequently we assess, over three simulation studies, whether the reduced MTL connectivity leads to 1) a deficit in selecting subordinate stimuli over dominant ones based on context information; 2) increased false alarms and intrusions in memory tasks and 3) deficits in the retrieval of context information. To asses the first hypothesis we implemented a lexical disambiguation task in which semantically ambiguous words are preceded by a context that primes either the dominant or the less frequent meaning. To test the second hypothesis we simulated a list learning task and for the third hypothesis a source monitoring task. The simulation results were compared with extant data. We also performed more formal analyses to assess how different pathways within the MTL contribute to context processing deficits in schizophrenia.

## Materials and Methods

### Model architecture

**Figure 1:**
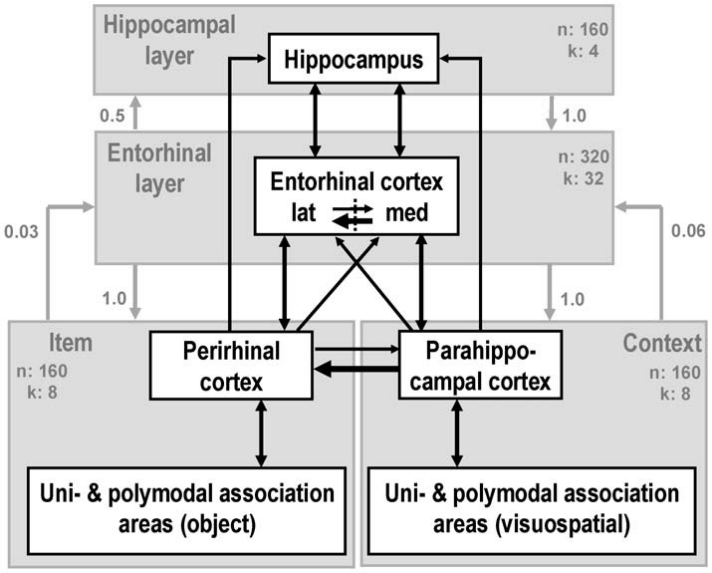
Diagram of the model used in the simulations. The white box model represents the simplified anatomy of the medial temporal lobe. The gray overlay depicts the four modules of the model. For each module, the number of nodes (n) and the global inhibition parameter (k) are shown. Model connections are depicted by arrows, with connection density (as a proportion of full connectivity) listed besides each connection.

Model architecture and technical implementation have been described previously [28] and were not altered to perform the current simulations. The model captures the basic organization of the (para)hippocampal regions in a simplified manner (Figure 1): two input modules, labeled ‘object’ and ‘context’, represent the perirhinal and parahippocampal cortices, processing object [41] and spatial information [42] respectively. Fanning projections from these modules converge onto the entorhinal module [39], [43], where the inputs are integrated. Plasticity of these connections is relatively low, so synaptic weights changed negligibly on the time scale of the simulations. The highest module in the hierarchy represents the hippocampus. It is connected to the entorhinal module through dense and highly plastic, reciprocal connections. Entorhinal feedback connections to the object and context modules produce the model output.

The context module has a denser projection to the entorhinal module than the object module. This architecture was motivated by functional and anatomical considerations. At the functional level, contextual information has a low rate of change, while item input changes at a faster pace. As a consequence the context module must have a denser projection to layers where pattern integration takes place than the item input, to support associations of an enduring contextual representation with multiple items. This is in line with anatomical evidence: parahippocampal and medial entorhinal cortex (context stream) project heavily to the perirhinal and lateral entorhinal cortex (object stream), while the inverse projection is much sparser [43]. To maintain a balanced influence of object and context input to the entorhinal module, the object projection has, on average, stronger weights of the individual connections. Thus, the context projection is dense with relatively week connections, while the object projection is more sparse, but with relatively stronger connections. In our model this architecture leads to some realistic memory characteristics. In particular, it enables many objects to be stored in conjunction with the same context and enhances the efficacy of context information to function as a search cue (context cues lead to a ‘broader’ search of the memory store than object cues).

The model uses linear threshold nodes. Such nodes simply add up incoming signals from other nodes and switch between an ‘inactive’ and an ‘active’ status, depending on a threshold activation value. The connections between nodes have modifiable weights, representing synapses. Learning in these connections was implemented with an asymptotic variant of the Hebb rule [44].

Global inhibition is mimicked by *k*-Winner-Take-All dynamics, which limits activity in a layer to a predetermined number of nodes (*k*) receiving the largest input. This ‘*k*’ is relatively large in the entorhinal, and small in the hippocampal layer, in accordance with electrophysiological data [39]. Noise was introduced in the model by randomly activating, at every iteration, a small number of nodes with a given probability. Parameter settings for the intact model, representing the system in healthy individuals, are shown in figure 1.

#### In silico neuropathology

**Figure 2:**
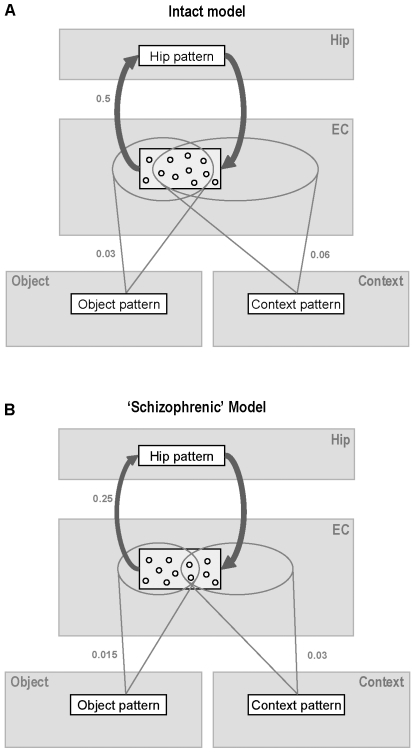
Integration of object and spatial information in the parahippocampal regions of the model. The four modules of the model are shown in light grey; the active patterns in the four modules are shown as white rectangles. Only the nodes making up the active pattern in the entorhinal module are depicted. Connection densities (expressed as a proportion of full connectivity) are given for the feedforward connections in the intact model (a) and the schizophrenic model (b). (a) In the intact model there is considerable convergence of input connections on entorhinal nodes (overlap area of projections from the active object and context patterns). Thus, when an object-context pairing is being learned, many entorhinal nodes get input from both the object pattern and the context pattern. However, reduction of the input projections (b) reduces the probability that a given entorhinal node receives input from both sources. This favors the inclusion of nodes receiving only context- or only object input in entorhinal representations. Since single object projections are stronger than single context projections, neurons receiving only object input have a higher chance of winning the competition for activation than neurons receiving only context input. Thus object information gets overrepresented in the entorhinal pattern, at the expense of context information. Due to this circumstance, object cues activate large parts of entorhinal patterns and can lead to retrieval irrespective of context cues. Conversely, isolated context cues activate only a small portion of associated entorhinal patterns, which is often insufficient for successful retrieval. EC: entorhinal cortex; Hip: hippocampus.

As in our previous paper [28], schizophrenia neuropathology was simulated by reducing the connections from the input layers (‘object’ and ‘context’) to the entorhinal layer, and the connections from the entorhinal to the hippocampal layer, by 50%. This is in line with studies showing substantial loss in the density of synaptic and dendritic molecules in the parahippocampal region and in hippocampal subdivisions targeted by the perforant path [26], [45], [46]. Figure 2 gives the resulting connectivity parameter values for the intact and ‘schizophrenic’ model.

To isolate the contributions of the two sets of connections to functional deficits, we also analyzed the effects of reducing just one of the two levels of connectivity (inputs-to-entorhinal, or entorhinal-to-hippocampus) by 50%. More technical details are given in Talamini et al., 2005 and, as supporting information, in Text S1 (section ‘Additional methods’).

### General functioning of the model

To simulate learning of an episode, a set of nodes is activated in the object layer and another set in the context layer. The patterns in the two input layers stimulate a set of nodes in the entorhinal layer. Out of this set, the nodes with the largest input become active, forming the entorhinal representation of the item-context co-occurrence. Some of the activated entorhinal nodes represent information from just one input layer, but most receive both types of input, and thus cross-associate the item and context patterns (Figure 2a). Similarly, the activated entorhinal nodes select a smaller group of hippocampal nodes. Through highly plastic, bi-directional connections between the entorhinal and hippocampal layer, these two representations are bound together, forming the episodic trace.

The memory system can be sampled using cues, consisting of partial input patterns; for instance, part of a context representation from a previously experienced episode. Initially, such a cue may activate only part of an associated entorhinal pattern, but if the set of activated entorhinal nodes sufficiently resembles a stored representation, their combined firing will tend to activate associated hippocampal nodes, through the previously strengthened connections with these nodes. The hippocampal nodes, in turn, will recruit missing nodes of the entorhinal representation. Over a number of cycles, this pattern completion process will reinstate the original pattern in the entorhinal layer, which, in turn, can reinstate associated information in the input layers, namely, item representations that have been experienced in that particular context (feature extraction). Thus, all features of an episode can be recalled, even when only one of the input layers is cued.

### Simulations

#### General procedures

Object and context representations consisted of eight nodes each, activated in the object and context layer, respectively. In all learning procedures synaptic transmission in the feedback connections of the hippocampal layer was dampened, so that the activity in the network was largely determined by the ‘on-line’ inputs [47], [48]. Learning then occurred over three iterations for each item-context pairing. Following learning, synaptic transmission in the feedback connections was restored, to allow the influence of feedback activity during subsequent retrieval sessions.

Before each simulation, weights were initialized to simulate the background of a ‘full memory’. To also simulate earlier encounters of the subject with the items used in the various experiments, we stored each object (including foils) in a random context before running the paradigm, using a variable learning parameter. We refer to this procedure as ‘prelearning’. Retrieval was thus tested under competitive circumstances.

Simulations were kept at a semi-quantitative level: parameter values were the same for all paradigms, and were not optimized to produce a best quantitative fit for the experimental data. All parameters listed in our previous paper [28] were kept at the same value. Where new parameters were introduced, mostly in our implementation of concrete tasks, we explored other values to ascertain that our results were robust against variations in these parameters, as described in Text S1. Each simulation was repeated at least 50 times to guarantee reliable results.

#### How are MTL representations altered in the schizophrenic model?

In neural network models such as this, the activity of each node can be tracked. How object and context information are processed and represented can thus be observed directly. We assessed the integration of object and context information in the intact and schizophrenic model, by presenting the model with one object and one context, and counting how many nodes in the emergent entorhinal pattern represented object information, how many context information and how many the combination of the two.

#### Contextual constraints in lexical disambiguation

To tests contextual constraints in selecting subordinate stimuli over dominant ones we implemented a lexical disambiguation task. Such tasks typically use homographs with a dominant (high-frequent) and subordinate (low-frequent) meaning. The homographs are embedded in a sentence that fits with either the dominant or the subordinate meaning of the homograph on semantic grounds (e.g., “The bank had just been robbed by bandits” and “The bank had just been planted with grass”). After each sentence, participants are asked to make some decision that reflects the accessibility of the two meanings of the homograph (e.g. ‘does the sentence make sense?’). If the sentential context guides which word meaning is retrieved, the participant will endorse the fit, but if the dominant meaning is retrieved independent of context, participants will make errors.

For our simulation we constructed four pairs of homographs, each pair consisting of two word meanings with the same word form. Only one homograph of each pair was used to complete a sentence in the simulation. In each pair, homographs shared 50% of their representation (four nodes representing word form), while the other 50% was unique to each representation (four nodes representing meaning). We varied the amount of prelearning that each homograph meaning received to simulate frequency of occurrence (for an explanation of prelearning see ‘General procedures’). High-frequent meanings were given five prelearning trials; low-frequent meanings were given two prelearning trials. Conditions were crossed such that one pair of homographs encompassed a high-frequent correct and a low-frequent incorrect meaning, one encompassed a low-frequent correct and a high-frequent incorrect meaning, with the remaining two consisting of two high-frequent meanings or two low-frequent meanings. Then, sentence comprehension was tested. First, we activated 6 of 8 nodes representing a random context and 3 of 4 nodes representing the word form of the homograph. After 20 iterations, 6 context nodes were activated that were part of one of the contexts in which the contextually correct homograph had been prelearned. This represented reading of the sentence part giving the context. Correct performance consisted of retrieval of the contextually correct homograph meaning within 130 iterations. Retrieval of the incorrect meaning was scored as an error and no retrieval as an omission.

#### Contextual constraints in memory

In typical episodic memory experiments, participants learn a list of items, usually objects, and then are asked to retrieve the learned material, for instance through free recall, cued recall or recognition. To implement list learning [28] the model was presented with a list of 10 objects. The object representations were activated one at a time, together with one stable context representation common to all the objects. Retrieval was then tested under conditions representing free recall, cued recall and recognition. In our simulation of free recall, a context cue was set by activating 75% of the pattern representing the learning context. Performance was measured as the number of different list objects retrieved in response to this cue over 150 iterations. Intrusions were scored when an object was retrieved that was not on the list.

In cued recall and recognition, the model was additionally provided with object cues. The recognition cue consisted in 75% of an object pattern. The cued recall cue consisted in half the recognition cue (representing, for instance, a word-stem or category cue). The recognition paradigm included presentation of foils (objects which did not occur on the studied list) at test, which had to be rejected. The foil items were unrelated to the list items. Hence, their representations in the item layer overlapped only randomly with those of list items. Retrieval of a pattern within 50 iterations after cueing counted as a recognition answer, while failure to retrieve a pattern counted as a ‘no recognition’ answer. Recognition answers were scored as hits when the cued object was a learned one, and as a false alarm when the cued object was a foil.

#### Retrieval of context information

In source monitoring experiments, participants are presented with items from two or more different sources, for instance different speakers, locations, or presentation media. Again, the items are often objects. In a later memory test, participants are shown items that were presented earlier and foil items that were not. They are asked whether a specific item was presented at study, and from which source the item originated [49].

To simulate source monitoring, the model was presented with 20 different objects in two alternating contexts. The object representations were activated one at a time, together with one of the two context representations, which overlapped by 50%, to simulate a partial difference in object presentation (e.g., objects presented by different voices in the same room).

During test, the 20 learned objects were presented sequentially, intermixed with 10 foils. Following each object presentation, the model was allowed to update its activity over 50 cycles, or until any context representation reached threshold. If activation of one of the context representations crossed threshold, the object was counted as assigned to that context; this could be either a correct attribution, or a misattribution. If neither context representation crossed threshold an omission was scored. If a foil object led to either context representation reaching threshold a false alarm was scored.

### Methodological considerations

The model makes several reasonable simplifications with regard to MTL anatomy. While higher order inputs onto the hippocampus are probably integrated over a number of steps, in the model these are taken together as one. Similarly, orthogonalization in the hippocampus appears to occur in two sequential steps, which in our model are condensed into one step. Although relinquishing some precision in the mapping of the model onto the real circuitry, these simplifications allow us to transparently implement the dual MTL features of integration of information, on the one hand, and storage with orthogonalization on the other hand. A discussion on the possible effect of these simplifications on model function is presented in the supporting information (Text S1, (section ‘Methodological considerations’).

It should also be pointed out that a comparison between the model's performance and the human data simulated in this study cannot be made at a quantitative level. First, due to fundamental differences between any neural network model and the biological circuitry being simulated (for instance with respect to capacity) the comparison of absolute performance scores is of limited value. Second, we chose to hold parameter values constant for all simulations, and thus forego attempts to fit particular data sets. Rather, model performance and human data were compared at the level of the within-group performance profile over multiple tasks. That is, the model and human memory show similarities with respect to relative performance on different tasks.

## Results

### How are MTL representations altered in the schizophrenic model?

The connections between the input layers and the entorhinal cortex serve the integration of object and spatial-configural information. The reduction of these connections in the schizophrenic model may lead to abnormalities in this process. To quantify the integration of object and context information in entorhinal patterns, we calculated the proportions of neurons in a pattern that receive input only from the active object pattern, only from the active context pattern, or from both input patterns. Only the latter neurons represent the combined occurrence of the information from the two sources, providing a good measure of integration.

As shown in Table 1, most entorhinal pattern nodes in the intact model indeed represent both input sources (79%). However, in the schizophrenic model only 25% of the nodes represent input from both sources; the level of integration is thus very low. Moreover, 56% of nodes only represent information from the object layer, compared to 20% that only reflect context input. Thus, the schizophrenic model is biased to represent object information at the expense of context information.

**Table 1:** Input integration in the entorhinal module.

	control	schizophrenia
only item inp	0.15	0.56
only ctxt inp	0.08	0.2
Both	0.79	0.25

Proportions of nodes in entorhinal module representations that receive input from only the active object, only the active context, or both sources.

To understand the origin of this processing deficit we must consider the anatomy of the intact model, in which the context stream has a denser projection than the object stream, with lower average weights (see ‘Model architecture’ for rationale). In the intact model, the connections from the object and context layer are dense enough to ensure that most nodes in the entorhinal layer receive input from both sources (Figure 2a). However, in the schizophrenic model, the reduced connectivity between input layers and entorhinal module leads to incomplete convergence of object and context input onto the entorhinal module (Figure 2b). Thus, many neurons receive input only from one source. As a result of the stronger object connections, nodes carrying only object information end up being more numerous in the entorhinal representations than nodes conveying only context input.

At the behavioral level this leads to context being a poor retrieval cue (e.g., in free recall), as context cues will often activate too few entorhinal neurons to enable successful pattern completion. When both object and context cues are available, (e.g., in recognition, source monitoring or lexical disambiguation) object cues will tend to override context cues. This occurs because the exaggerated proportion of entorhinal nodes carrying object information has an overly large influence on pattern completion. As a consequence, strongly represented objects get retrieved even when this is contextually inappropriate. In such cases the appropriate context is not retrieved with the object.

The reduction of entorhinal efferents to the hippocampal module also leads to processing problems. As explained in our previous paper, this pathway enhances pattern separation in the system. This means that patterns that overlap to a certain degree at the entorhinal level, overlap to a much lesser extent at the hippocampal level. Thus, patterns can be retrieved separately.

The reduction of the entorhino-hippocampal connection causes decreased pattern separation in the system. In fact, mean overlap between hippocampal patterns is increased from 6% to 13%. The overlapping parts of hippocampal representations are connected to the entorhinal representations of multiple object-context pairings. In some cases this can lead to omissions in retrieval because none of the competing entorhinal patterns is sufficiently reactivated to reach threshold (e.g. in recognition paradigms, as shown in Talamini et al, 2005). However, increased overlap can also contribute to retrieval of inappropriate patterns, especially in paradigms in which the retrieval cues co-activate strongly encoded alternative patterns (e.g. in source monitoring, see below).

### Contextual constraints in lexical disambiguation

Table 2 shows the results from the lexical disambiguation simulation. Since the performance scores on sentences containing a homograph with 2 low-frequent meanings or a homograph with two high-frequent meanings were very similar, the results for these two conditions were averaged (‘equal frequency’ row in table 2). While the schizophrenic model displays some level of deficit in all conditions, the largest appears in the condition in which sentence context primes the subordinate (low-frequency) meaning of the homograph. In this case, the schizophrenic model shows a disproportionally increased tendency to activate the contextually incorrect, dominant homograph meaning. This suggests a reduced influence of the contextual information on retrieval of word meaning.

**Table 2:** Proportions of retrieved patterns in the three simulations.

simulation		control	schizophrenia	input-to-EC	EC-to-hip	SEM
lexical disambig-uation	strong vs. weak	0.88	0.81	0.76	0.85	0.04
	equal strength	0.77	0.67	0.55	0.74	0.03
	weak vs. strong	0.7	0.34	0.4	0.55	0.05
list learning	free recall	0.49	0.21	0.14	0.42	0.03
	intrusions	0.02	0.02	0.01	0.03	0.01
	recognition hits	0.88	0.74	0.81	0.80	0.01
	false alarms	0.09	0.16	0.21	0.10	0.01
source monitoring	source hits	0.58	0.42	0.23	0.53	0.02
	source errors	0.007	0.033	0.007	0.028	0.002
	omissions	0.41	0.55	0.77	0.44	0.02
	false alarms	0.024	0.086	0.016	0.061	0.01

For the lexical disambiguation simulation, the proportion of correct meaning retrieval is shown (i.e. the meaning consistent with the sentence context), in conditions in which it was the dominant of two homograph meanings (‘dominant’), the subordinate of the two (‘subordinate’), or one of two homograph meanings of equal frequency (‘equal frequency’). In the list learning simulation, free recall and recognition refer to the retrieval of list objects during a free recall or recognition test. Intrusions are recalled patterns that were not part of the studied list, while false alarms are foils falsely endorsed during the recognition test. In the source monitoring study, what is retrieved is either the correct source (source hit), the incorrect source (source error), or nothing (omission). False alarms here refer to the retrieval of a source for a foil object that had not been presented at study. Data are given for the intact model, the model with reduced input-to-entorhinal module connections (input-to-EC), reduced entorhinal module-to-hippocampus connections (EC-to-hip), or both sets of connections reduced (schizophrenia). To render an idea of the reliability of the model data, the standard error of the mean (SEM) is given for the scores pertaining to the normal model.

**Figure 3:**
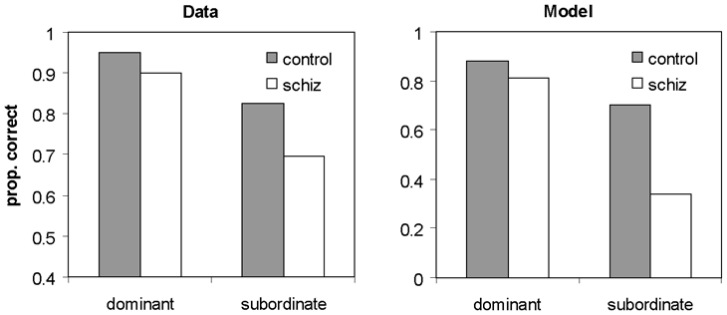
Lexical disambiguation: experimental data and simulation results. Retrieval of the meaning of polysemic words, cued with a sentence that primes either the dominant or the subordinate meaning of the word. In the left panel data from a lexical disambiguation task, in which participants are given a sentence containing a polysemic word, and have to judge whether the sentence makes sense. Negative judgments are taken to indicate a failure of correct meaning retrieval (Salisbury et al. [7], figure 1, values for dominant and subordinate condition). In the right panel: data from model simulation.

As shown in figure 3, the pattern of model performance closely mimics findings in a lexical disambiguation experiment in humans [7]. In this specific experiment, participants were presented with sentences containing a homograph. Half of the sentences affirmed the dominant meaning of the homograph, one-half the subordinate meaning (an additional set of sentences contained a noun with one meaning). After each sentence, participants were required to indicate as quickly as possible whether or not the sentence made sense to them by pressing one button for ‘sensible’, or another for ‘nonsensical’. The right panel of figure 3 shows the proportion of correct responses (‘sensible’ responses) to each type of sentence (affirming the dominant meaning and affirming the subordinate meaning of the homograph). As can be seen, both groups perform better on comprehending dominant homograph meanings and patients make more errors than healthy participants to all sentences. However, patients make disproportionately more errors than controls to subordinate sentences.

Simulations in which just one level of connectivity is reduced (Table 2) show that the deficit is largely due to changes in the lower level of connectivity, from the input layers to the entorhinal module.

### Contextual constraints in memory

Table 2 gives the rates of correct free recall, intrusions, recognition hits and false alarms, for all model configurations. As reported previously [28], the performance profiles for the intact and schizophrenic models on recall and recognition are in line with data from healthy participants and patients with schizophrenia [29]. The rate of intrusions in recall is low in the intact model, with slightly more false alarms in recognition. In the schizophrenic model, the false alarm rate is substantially increased. Intrusion rates are about the same in the intact and schizophrenic models, but were much increased relative to correct recall in the schizophrenic model.

We compared model performance to experimental data reported by Elvevåg and colleagues [12]. In this study, participants were presented with a list of words, followed by a free recall and a recognition test (the study differentiates between errors related and unrelated to list items; only unrelated intrusions and false alarms are considered here). As in our model, the absolute number of intrusion errors in free recall was similar in healthy controls and patients with schizophrenia (0.94 intrusions in healthy participants vs 0.84 in patients). However, correct recall in patients with schizophrenia was reduced by about 50% with respect to healthy controls (7.59 vs. 14.74). Thus, when examining the number of intrusions as a proportion of overall memory (i.e. the number of correctly recalled items), patients performed much more poorly than controls. These findings are in accordance with our model, in which the number of intrusions in the intact and schizophrenic model is also similar (0.2 in both cases), but recall is reduced by 57% in the schizophrenic model with respect to the intact model (see Table 2).

False alarm rates in recognition (proportion of foil items that is falsely recognized) were about 50% higher in the Elvevåg study for patients than for healthy controls (0.12 vs. 0.19, computed on the basis of their Table 3). This was also true in the model (see Table 2). Thus, false alarm rates are increased to a similar extend in patients and in the schizophrenic model.

**Figure 4:**
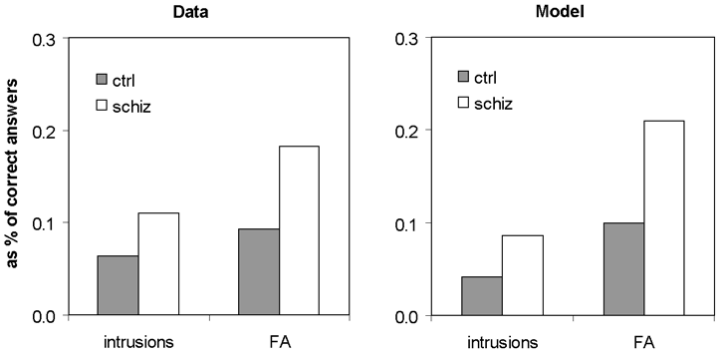
False alarm production: experimental data and simulation results. Intrusions in a free recall task as a proportion of correct recall, and false alarms in a recognition task as a proportion of correct recognition. Left panel: data from a standard list learning study (Elvevåg et al. [12], table 3. For recall intrusions, ‘Errors unrelated to studied list’ were divided by ‘Correct’; for recognition false alarms, ‘Unrelated false alarms’ were divided by ‘Hits’); right panel: data from model simulation.

Figure 4 shows intrusions and false alarms, in humans and in the model, as proportions of overall retrieval (i.e. number of intrusions/number of correctly recalled items; number of false alarms/number of correctly recognized items). The model observations closely mimic findings in patients.

Simulations with only one level of connectivity reduced (Table 2) show that the increased false alarm rate is due to the reduction of the lower level of connectivity, from the input layers to the entorhinal module.

### Retrieval of contextual information

Source-monitoring in the model was compared to a controlled patient study by Vinogradov and colleagues [18]. In this study participants read aloud 20 experimenter-generated words, and 20 self-generated words, in an alternating fashion. They were later shown these words, among new foil words, and were asked to determine whether each word was self-generated, experimenter-generated, or brand new.

**Figure 5:**
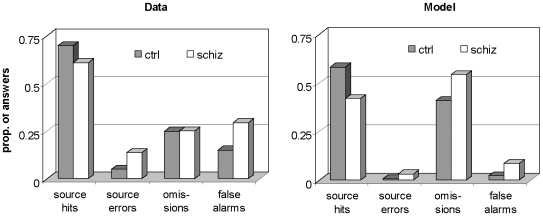
Source monitoring: experimental data and simulation results. Source monitoring data, split out in retrieval of the correct source (source hit), the incorrect source (source error), or nothing (omission). False alarms refer to the retrieval of a source for a pattern that has not been presented at study. Left panel: Data from a study of Vinogradov and colleagues [18] (table 3. For source hits: correct attributions ‘Experimenter’+correct attributions ‘Self’/40; for source errors: misattributions ‘Experimenter’+misattributions ‘Self’/40; for omissions: 60 - sum of all responses/60; for false alarms: misattributions ‘New’ divided by 20). Right panel: data from model simulation.

Figure 5 shows participants' performance and data from the concomitant simulation. The proportion of list items that are attributed to the correct source is given as ‘source hits’; the proportion of incorrect attributions as ‘source errors’. ‘Omissions’ are list items for which no source is retrieved and ‘false alarms’ is the proportion of foil items attributed as either self-generated or experimenter generated. In comparison to the human data, the model produces fewer hits and errors, and more omissions. This may be due to guessing on the basis of partial information, which is possible for human participants but not in the model. However, the deficit pattern in the schizophrenic model is very similar to that in patients with schizophrenia: with respect to the intact model correct responses were reduced, omissions were slightly increased, while errors and false alarms were substantially increased.

The two levels of connectivity contributed differently to the source-monitoring deficits (Table 2): reduction of inputs to the entorhinal layer led to a drastic reduction in hits, a milder decrease in false alarm rate and a somewhat increased omission rate. Thus, to some extent there is an overall response reduction. On the other hand, reduction of entorhinal efferents to the hippocampal module underlies the increase in source misattributions and false alarms.

### Summary of results

In general the MTL model performs somewhat below the level observed in human experiments and the deficits in the schizophrenic model are somewhat exaggerated with respect to the observations in patients. However, relative performance of the model over different task conditions, as well as the relative pattern of deficit displayed by the schizophrenic model (i.e. dysfunction as a proportion of normal function), closely resemble performance profiles in healthy participants and schizophrenic patients on all simulated tasks. Simulations reported in the supporting information (Text S1, section ‘Parameter settings’) show that this pattern of results was not due to our choices in implementing the tasks, but that it was also found when other choices were made.

The two connectivity levels contribute differently to the evaluated dysfunctions: a lack of contextual constraints, both in lexical disambiguation and in the list learning task, is largely due to the reduced connectivity between the input layers and the entorhinal cortex. This same reduced connectivity underlies a retrieval deficit for context information in a source monitoring task. These deficits can be understood as consequent to the unbalanced representation of object and context information, as well as the poor integration of the two, which are both induced by the reduction of this set of connections.

The reduction of entorhinal efferents to the hippocampal module contributes to functional deficits, causing an increase in source misattributions and false alarms in the source monitoring task. This occurs, because the increased overlap induced by the reduction in this set of connections enhances formation of strong attractors that, over time, get linked to an increasing amount of inputs and outputs. Through such patterns, sometimes called ‘spurious attractors’, object cues may activate the wrong context pattern, leading to source misattributions. Even foil objects may activate a spurious pattern, which in turn might activate one of the learning contexts, leading to false alarms.

The formation of spurious attractors occurs especially in paradigms in which similar stimuli are repeatedly presented; for instance in source monitoring.

## Discussion

Context processing in schizophrenia was evaluated using a computational model of the MTL. The neuropathology observed in schizophrenia, implemented in the model, led to severe context insensitivity, associated with impairments in choosing subordinate over dominant responses based on context cues, high false alarm rates in object recognition and deficient retrieval of context information in source monitoring. The deficit patterns in these tasks closely resemble the ones observed in patients with schizophrenia [7], [12], [18].

We have demonstrated that these deficits may originate from reduced connectivity in parahippocampal regions, leading to formation of abnormal episodic representations, with poor binding of object and spatial features and a predominance of object information, at the expense of spatial information. Due to this bias object cues tend to override context cues, which place only weak constraints on MTL processing. In addition, the poorly integrated entorhinal representations lead to reduced retrieval in paradigms that strongly depend on links between the different aspects of an episode, for instance source monitoring and free recall.

As we have shown in our current and previous studies [28], a second factor contributes to the behavioral deficits: the reduced entorhinohippocampal connectivity leads to increased representational overlap in the system. This means that some patterns are not stored in a distinctive episodic representation and cannot be retrieved at all. On the other hand, increased overlap enhances formation of so-called spurious attractors: strong patterns, connected to many inputs and outputs. Such patterns may get activated inappropriately; especially in paradigms that enhance formation of overlapping representations through presentation of similar stimuli (e.g., source monitoring).

These findings suggest that at least some of the context processing deficits in schizophrenia may originate from MTL pathology. We have argued previously that the same neuropathology also underlies central memory deficits associated to the disorder [28]. We therefore suggest that episodic memory and context processing impairments in schizophrenia reflect the same underlying binding deficit.

This notion is in line with broadly accepted theories stating that the hippocampus serves conjunctive coding of stimulus features [23], [50]. This includes conjunctions between object identity and position [36], [37], [39], as well as temporal conjunctions [51], [52]; in other words, the processing of stimuli in a spatiotemporal context. The current model emphasizes the role of the parahippocampal region in conjunctive coding, suggesting that it serves the successful integration and balanced representation of information from different hippocampal input sources.

It seems reasonable to assume that supramodal MTL representations partly guide our associations and train of thought. If so, the tendency to over-represent objects at the expense of context would gear patients' associations and responses towards objects. Conversely, the current context would have little influence on the patient's train of thought. This may lead to symptoms such as concreteness, tangentiality (straying from context in discourse) and contextually inappropriate behavior.

In addition, the tendency to activate spurious attractors may bias associations towards stored memories. More specifically, with the environmental context continuously being neglected, isolated object cues may activate spurious patterns. Context information associated to the spurious patterns may then be brought “on line”, overriding the weak activation from the “real” context. This could manifest itself in the preoccupation with persistent delusional themes, seen in many patients with schizophrenia.

As our findings illustrate, MTL abnormalities may lead to deficits in tasks that are not typically considered episodic memory tasks, but that nevertheless would require processing of stimuli in the context of spatially or temporally distal information. For instance, language processing. While a sharp dissociation has often been drawn between semantic and episodic memory, with the hippocampus only involved in the second, several recent studies suggest a more nuanced view (for discussions see [53], [54]. These studies suggest that the hippocampal region contributes to language comprehension by processing relations between the different words and concepts making up a message. Indeed, activation of the hippocampus and parahippocampal regions occurs during semantic tasks that require extensive contextual processing [55]–[61] and MTL damage leads to dysfunction on such tasks [54], [62]–[65], for instance, in resolving sentential ambiguity [63], [64], [66].

Interestingly, subtle language impairments described in relation to schizophrenia resemble the ones seen after MTL dysfunction, suggesting a similar neural origin. Specifically, analyses of patients' speech have demonstrated reduced cohesion, complexity and hierarchical organization [67], as well as increased incidence of syntactic errors and dysfluencies [68]. Such deficits might originate if past discourse elements do not sufficiently constrain the choice of current ones. Furthermore, unusual associations have been reported [69], as well as abnormalities in processing sentential ambiguity. In line with our model, both latter abnormalities appear to result from reduced sensitivity to context and a preference for the dominant meaning of polysemic words [70]–[72]. An extensive review [73] concluded that language competence appears to be intact in schizophrenia, and that the language problems reflect more general problems in information processing. We suggest that the type of binding deficit described in our model is a likely candidate.

There are also alternative theories regarding impaired context processing in schizophrenia. The central ideas in these theories are either that prefrontal dysfunction leads to a deficit in *maintaining* context information in memory [3], [74], or that there is something wrong with (prefrontal) inhibitory mechanisms, so that context information is *not used* for inhibitory control [71]. These theories have substantial overlap with our own, especially regarding the way in which contextual processing deficits are thought to affect widespread cognitive functions. However, in contrast to other theories our model proposes that adequate representations of stimuli in their spatiotemporal context are never *formed* to begin with and that the MTL region strongly contributes to this problem. (Note that our model does not exclude deficient maintenance and use of poorly bound contextual representations in later mental operations.) Thus, our model predicts that problems using contextual information should occur as soon as the stimulus reaches higher brain areas, including the MTL cortex. That is, a few hundred milliseconds after stimulus onset. Studies are underway to test this prediction.

In support of our model, several recent studies suggest a binding deficit in schizophrenia. Some of these show that memory deficits in schizophrenic patients are most severe when performance depends entirely on binding of features. This is the case in associative recognition, in which pairs of objects are pitted against recombinations of those objects, rather than against novel foils. In two studies using this paradigm performance of patients with schizophrenia actually dropped to chance level [13], [21].

In other studies, memory for objects was assessed in conjunction with memory for source, temporal or spatial information [14], [15], [21]. In these studies, patients with schizophrenia show significant deficits in remembering contextual information associated to objects, independently of memory for the objects themselves. One such study [14] assessed both source and temporal information associated to objects: while in healthy controls recognition of objects was usually accompanied by correct source and temporal judgments (about three quarters of the time), patients with schizophrenia could identify the correct source and temporal context for only 40% of the objects they recognized. The results indicate a deficit in binding the different elements of an episode together, independent of performance on object recognition. These effects held true for subgroups of patients and controls that were matched for performance on object recognition, indicating that the impairment is not related to poor object recognition per se, but is specific for the schizophrenia pathology.

The notion of a binding deficit in schizophrenia is thus gaining support from an increasing body of evidence. Conversely, the altered balance between object and context processing predicted by our model is a novel notion, which has not yet been tested directly (although there are some interesting studies suggesting that object and spatial processing are differentially affected by schizophrenia in other cognitive domains, including visual perception[75], mental imagery[76] and working memory[75]). Specific predictions derived from this notion include the following: 1) intra-object cues should be relatively more effective than context cues in eliciting retrieval in schizophrenic patients; 2) the normal benefit from context cues on retrieval should be reduced in schizophrenia (i.e., when the context changes from learning to retrieval, this should affect memory less in patients with schizophrenia than in healthy controls, and vice versa); 3) in processing language, patients with schizophrenia should be relatively insensitive to semantic violations at the discourse level (i.e. contextual inconsistencies), as long as individual objects featured in the discourse are highly associated.

We have recently verified the second prediction, showing reduced sensitivity of retrieval to context cues in schizophrenic patients (paper in preparation). Studies are ongoing to test the other predictions.

It might at this point be noted that, without further specification, context processing is a very general construct that likely involves different brain areas depending on the type of information being processed, and the period over which it is maintained. Accordingly, we do not mean to imply that our model captures schizophrenia impairments on all tasks associated to contextual processing, nor that MTL pathology is the sole source of behavioral problems in schizophrenia. However, the processing deficits we have described may have a profound influence on performance in tasks that rely on linking multimodal stimuli over time and space.

As a further point of consideration, source monitoring studies in schizophrenic patients, including the study by Vinogradov and colleagues [18], [22], have often found a strong tendency in the patient group to attribute self-generated information to an external source; a phenomenon that some relate to the occurrence of hallucinations. While such a response bias may indeed be present, the basic results of these studies were also found in experiments in which self-reference was not involved [13], [15]. Our results suggest that source-monitoring deficits are part of a fundamental episodic memory impairment in schizophrenia. Indeed, source monitoring deficits, like other memory impairments in schizophrenia, appear to be stable over time, resistant to drug therapy, and not attributable to attentional or executive factors [18], [77].

Our model has interesting correspondences to other network models on schizophrenia [78]–[81]. Indeed, some more abstract models have also suggested that spurious attractors, induced by reduced connectivity, may contribute to dysfunctions in schizophrenia [79], [80]. Furthermore, a recent model addresses how reduced NMDA receptor function would affect processing over particular (para)hippocampal pathways [81]. In line with our own work, this model suggests that context-dependent retrieval deficits in schizophrenia originate in the (para)hippocampal region.

In conclusion, we have developed a computational model that specifies the role of the MTL in contextual processing and episodic memory. According to this model, reduced parahippocampal wiring induces an information processing ‘syndrome’ characterized by poor binding of event components, with a predominant representation of object information at the expense of context information. In addition, there is reduced pattern separation with formation of spurious attractors. We have shown that this leads to a loss of contextual constraints in information processing, which is manifest in various domains of cognition. As demonstrated in our current and previous studies [28], the deficit profile over multiple context processing and memory tasks displayed by the model closely matches the one observed in patients with schizophrenia. This strongly suggests that a parahippocampal disconnection pathology may underlie these cognitive deficits. Moreover, the same neuropathology may contribute to symptoms, such as memory intrusions, tangentiality, concreteness, contextually inappropriate behavior and delusions.

These findings provide new insights into cognitive impairment in schizophrenia. They stress the importance of MTL pathology as an underlying cause, suggesting a role that is not limited to long-term memory, but affects the way in which events are perceived in the first place.

## Supporting Information

Text S1 This document contains additional information about the model presented in Talamini et al, submitted to PLoS ONE. It contains additional implementational details (“Additional methods”), simulations that explore the sensitivity of the model to our design choices (“Parameter settings”) and some technical discussion remarks (“Methodological considerations”).
(0.12 MB DOC)
