# Supplementary material for: Dominance of Objects over Context in a Mediotemporal Lobe Model of Schizophrenia
Source: PLoS One. 2009 Aug 4;4(8):e6505. doi: 10.1371/journal.pone.0006505 (PMC2714963; doi:10.1371/journal.pone.0006505)
Supplement: Text S1 — This document contains additional information about the model presented in Talamini et al, submitted to PLoS ONE. It contains additional implementational details (“Additional methods”), simulations that explore the sensitivity of the model to our design choices (“Parameter settings”) and some technical discussion remarks (“Methodological considerations”). (0.12 MB DOC) [file pone.0006505.s001.doc]

# Supporting information to Talamini & Meeter (PLoS ONE)

Talamini & Meeter

This document contains additional information about the model presented in Talamini et al, submitted to PLoS ONE. It contains additional implementational details (“Additional methods”), simulations that explore the sensitivity of the model to our design choices (“Parameter settings”) and some technical discussion remarks (“Methodological considerations”).

## Additional methods

### Model neuron

The model is built with linearly summating nodes with k-Winner-Take-All dynamics. A node i can either be active (S_i_=1) or inactive (S_i_=0). Whether or not neuron *i* is active depends on its total input H, the weighted sum of the input it receives from all nodes *j* to which it is connected:

Equation 1: $H_{i}=\sum_{j} w_{ij}S_{j}$

The weights *w_ij_* can vary between 0 and 1. In each module, the *k* nodes with the highest total input (*H_i_*) become active. If several nodes on the cut-off have an equal total input, a random selection of these nodes is activated so as to keep the total number of active nodes equal to *k*. The parameter *k* is set separately for each module in the model (values are given in Figure 1 of the article).

### Learning rule

The learning rule used is the Oja variant of the Hebbian learning rule, which shows a good fit to long term potentiation (LTP) data [1,2]. The rule is given in Equation 2.

Equation 2: $\Delta w_{ij}=\mu(S_{i}S_{j}(1-w{}_{ij})-S_{i}(1-S_{j})w{}_{ij})$

As in the normal Hebbian rule [3], a weight is strengthened whenever both the presynaptic and the postsynaptic nodes fire (i.e., whenever *S_i_S_j_* is 1). The weights do not grow boundlessly, however: by multiplying weight change by 1*-w_ij_*, the Oja rule assures that with continuing learning weights asymptotically approach the implicit maximum value of 1. Weights decrease whenever the presynaptic node does not fire, while the postsynaptic node does (i.e., whenever *S_i_*(1-*S_j_*) is 1), modeling heterosynaptic long term depression (LTD). Again, weights do not decrease without bounds, but asymptotically approach the implicit minimum weight value of 0. Learning is scaled by the learning rate, *μ*, set separately for each connection (table 1).

Table 1. Mean initialization weights (with between brackets standard deviations)

and learning rates for all connections in both the intact model (Intact) and the model with

reduced connectivity (Schizophrenia).

### Weight initialization

An important characteristic of the Oja rule is that the long-term expected value of a weight is equal to the likelihood that a presynaptic node is active, given that the postsynaptic node is active [2]. For example, if the presynaptic node is active 60% of the times that the postsynaptic node is active, then the weight on the connection between the two nodes will tend to hover around 0.60. We use this characteristic to initialize the weights in most connections in our network. We initialize them to the value they would have after many independent patterns are learned. This simulates the background of a “full memory”.

The likelihood that a given pre-and postsynaptic node fire simultaneously depends on network connectivity. They are therefore different in the schizophrenia simulation than in the normal condition: some of the connections have higher mean initial weights in the schizophrenia simulation. This is because in the schizophrenic condition the same amount of information as in the normal model is routed over a reduced number of connections. Therefore, synapses undergo LTP relatively more frequently. To some limited extent this compensates for the processing deficit in the circuitry: in a simulation in which the initial weights in the schizophrenia condition were not adapted, but set to the same values as in the normal model, memory performance was worsened (data not shown).

Another obvious determinant of the likelihood that presynaptic node *x* fires given firing of postsynaptic node *y*, is whether there is a reverse connection between the two. If the presynaptic node gets feedback input from the postsynaptic node, the likelihood of simultaneous firing is higher than when no such reciprocal connection exists. Additional table 1 therefore lists weights of feedback connections separately for those node pairs that are reciprocally connected, and those that are not.

In all cases, the expected weight of a connection is calculated, using the binomial distribution, bottom-up determination of firing patterns during learning, and independence of input nodes. Although in this way the expected weights can be calculated, it is impossible to analytically derive the full weight distribution around each mean weight. Using Monte Carlo simulations, we determined that the shape of these distributions depends both on the mean value and on the learning rate. If the mean was not too low and the learning rate not too high, the distribution was approximately normal. We estimated the standard deviation of each normal distribution, and then added continuously distributed noise with the estimated standard deviation and a mean of 0 to the weights.

The one exception to this initialization procedure concerns the entorhinal-to-hippocampal projection, which was initiated at a higher value than the one calculated through the analyses detailed above. In the Oja learning rule, this alters the balance between LTP and LTD, favoring LTD over LTP. This was done to enhance the formation of orthogonalized patterns, as LTD favors pattern orthogonalization, while LTP diminishes it [4].

### Pre-learning

Prior to list learning, all items used in a simulation (including foil items) were learned with a random context to simulate recent exposures to these items. This was done with exponentially varying learning rates to simulate heterogeneity in item frequency and recency. The same was not done for list contexts, assuming that a context represents a unique configuration of stimuli. In combination with the weight initialization procedures described in the previous section, pre-learning allowed us to simulate the learning of new patterns against the background of a ‘full memory’, and test retrieval under competitive circumstances.

### Threshold setting

The input regions of the model also function as output regions. For example, activity in the item layer determines whether an item is retrieved or not. The output of the model is based on the feedback signal that the input regions receive from the entorhinal module. We assume that the input nodes have adapted to the statistics of the feedback that they receive from the entorhinal module. The threshold is therefore set at an optimal value in the interval between the expected feedback elicited from the entorhinal module if the pattern is retrieved, and the expected feedback elicited when a random collection of entorhinal nodes is active. This expected feedback differs for different permutations of the model. Therefore, thresholds were established for each permutation.

To set the criterion, we first we derived the expectations of two distributions of feedback to an Item node (see below for derivation): (1) the expected feedback given that an entorhinal pattern is active, which is associated with the right item, and (2) the expected feedback given that a random pattern is active (this could be either a pattern which has not been stored, or one associated with another item). Then, the criterion for retrieval was set at 50% of the interval between these two expected feedback signals. If the feedback signal to the item nodes exceeded this criterion, this is taken as evidence that a stored pattern has become active in the entorhinal module.

Because activating item nodes changes their feedback signal, the feedback signal was only measured in not-activated nodes. For example, in recognition, where 6 item nodes were activated as a cue, only the feedback signal to the two non-activated item nodes contributed to the output measure. We then averaged feedback to these non-activated item nodes, and count the item as retrieved if the average feedback exceeded criterion.

### Derivation of feedback expectations

The expected feedback to an Item node is equal to the number of nodes active in the entorhinal layer, multiplied by the expected feedback weight from each active entorhinal node to the Item node. The number of active nodes in the entorhinal layer is always equal to *k_ec_*, which leaves the distribution of weights as the sole determiner of the expected feedback. We will here derive the expected weight for the two cases mentioned above: the case that a random pattern is active in the entorhinal layer, and the case that a retrieved pattern, associated with the Item nodes under consideration, is active in the entorhinal layer. Both derivations will rely heavily on an already mentioned characteristic of the Oja rule: the expected value of a weight is equal to the likelihood that the presynaptic node is active given that the postsynaptic node is active.

A completely random entorhinal node has a likelihood of being active equal to *k_ec_/n_ec_*, the number of active entorhinal nodes divided by the total number of entorhinal nodes. The weight on the connection of a random entorhinal node to a random Item node will therefore hover around *k_ec_/n_ec_*. In the situation that a random pattern is active, the expected feedback to a single Item node will therefore be:

Equation 3 E(feedback | random pattern) = w_random_ * *k_ec_* = *k_ec_/n_ec_* * *k_ec_*

where w_random_ is the feedback weight from a random entorhinal node. As *k_ec_* is 32 and *n_ec_* is 320, this formula leads to an expected feedback of 3.2, in the case of a completely random entorhinal pattern.

The calculations for the expected feedback when a retrieved pattern is active in the entorhinal layer are much more complex. They must take three factors into account:

1) The fact that entorhinal nodes receiving feedforward connections from the active Item nodes will have a greater likelihood to be within the entorhinal pattern than entorhinal nodes that do not receive such connections

2) The fact that the pattern is learned

3) The fact that the pattern was prelearned.

Let us consider the feedback that one particular Item node receives from an entorhinal pattern that codes for an input pattern that the Item node is part of. The Item nodes in the pattern determine, together with context nodes, which entorhinal nodes will belong to the entorhinal pattern and which will not. Since each active item node is thus one of the determiners of firing in the entorhinal pattern, many nodes in the entorhinal pattern will be innervated by our Item node. The likelihood that an entorhinal node is part of the entorhinal pattern is thus higher when it receives a feedforward connection from our Item node, than when it does not. This, in turn, has consequences for the expected feedback weight: as explained above, feedback weights are higher when an entorhinal node receives a feedforward connection from the Item node, than when it does not. Thus, the formula for the expected feedback from retrieved entorhinal patterns to our Item node must take into account the different feedback weights from entorhinal nodes that receive a feedforward connection and those that do not:

Equation 4 E(feedback | retrieved pattern) = w_ffwd_ * k_ffwd_ + w_no ffwd_ * k_no ffwd_

where w_ffwd_ is the expected weight from an entorhinal node that receives a feedforward connection from the Item node, w_no ffwd_ is the expected weight from an entorhinal node that does not, k_ffwd_ is the expected number of entorhinal nodes in the pattern that receive a feedforward connection, and k_no ffwd_ is the expected number of entorhinal nodes in the pattern that do not (k_ffwd_ and k_no ffwd_ must sum to *k_ec_*). All four quantities can be found by calculating two interdependent conditional probabilities using the binomial distribution: k_ffwd_ and k_no ffwd_ are found via the likelihood that an entorhinal node is active given that it receives a feedforward connection from the Item node; w_ffwd_ and w_no ffwd_ are found via the likelihood that a node is in the pattern, given that it receives a feedforward connection. Both likelihoods depend on the value of *k_ec_* and on the connection parameters (in the intact model, we find the following values: w_ffwd_ = 0.484; w_no ffwd_ = 0.088, k_ffwd_ = 4.64, k_no ffwd_ = 27.36).

Equation 4 does not yet take into account the learning that takes place during the list-learning phase and the pre-learning phase. Accounting for the list-learning phase is straightforward. Since all nodes concerned are active in the list-learning phase (when the pattern is formed), a factor *α_l_(1-w)* (see Equation 2) is added to all expected feedback weights in Equation 4:

Equation 5 E(feedback | retrieved pattern) = [w_ffwd_ + α_l_(1- w_ffwd_)]* k_ffwd_ + [w_no ffwd_ +
α_l_(1- w_no ffwd_)]* k_no ffwd_

where *α_l_* is the learning rate in the learning phase.

The fact that patterns are also presented, with a random context, in a pre-learning trial, raises the expected feedback some more. As the pre-learning phase pattern does not overlap perfectly with the pattern in the learning phase, “unlearning” must also be taken into account (see Equation 2). It can be shown that, with progressively more learning trials in random contexts, feedback weights will exponentially approach the mean overlap between two pre-learning patterns (i.e., two patterns with the same item, but learned in different contexts). We thus approximate the expected weight after pre-learning as a step towards that asymptotic weight, *w_pl-asy_*, and add a factor *α_pl_(w_pl-asy_-w*) to each expected feedback weight, where *α_pl_* is the mean learning rate during pre-learning. Moreover, the fact that pre-learning takes place before the learning phase changes the expected weights at the outset of the learning trial. This changes how much learning can be expected to take place during the learning phase. Thus, the final formula for the expected feedback in the case of a retrieved pattern becomes:

Equation 6 E(feedback | retrieved pattern) ≈ [w_ffwd_ + α_pl_(w_pl-asy_- w_ffwd_) + α_l_(1- w_ffwd_ +
α_pl_(w_pl-asy_- w_ffwd_))] * k_ffwd_ + [w_no ffwd_ + α_pl_(w_pl-asy_- w_no-ffwd_) + α_l_(1- w_no ffwd_+
α_pl_(w_pl-asy_- w_no-ffwd_))] * k_no ffwd_

Here, *α_pl_* is 0.66*α_l_ (as learning parameters were drawn from an exponential distribution with *λ*=1.5 for pre-learning), and *w_pl-asy_* is 0.478. The whole formula, with all values given above, yields an expected feedback of 5.68. If the stored pattern associated with the item is active in the entorhinal layer, the item nodes associated with the item thus receive, on average, a feedback of 5.68. The criterion in the recall tests is set halfway this expected feedback, and the expected feedback when a random pattern is presented (3.2; see above) at 4.44.

There is one last complication in the recognition condition. Because activating item nodes changes their feedback signal, the feedback signal is only measured in not-activated nodes. However, the fact that the cued item nodes have been activated together with the not-cued item nodes in the pre-learning trial changes the feedback signal to these latter nodes (in this case, from 3.2 to 3.44). The criterion in recognition is therefore set with respect to the baseline feedback signal when the item cue is activated: 3.44 + 0.50*(5.68-3.44) = 4.56.

Because of different feedforward connectivity, feedback weights in the simulated schizophrenia model are different from those in the normal model (see Table 1). Expected feedback for items not stored remains the same (3.2), but the expected feedback from stored patterns increases (from 4.56 to 6.13). This leads to different threshold criteria from those in the normal model: 0.146 in recall, 0.1501 in recognition. As mentioned before, we repeated the simulation without these adaptations (i.e., with the same weight distributions and criteria as used in the normal simulation), and found that it produced the same pattern of result, but with worse performance in the schizophrenia condition.

## Parameter settings

To fit data from a set of experiments, one usually has to change parameter values from simulation to simulation. This makes sense: every study has a different participant group, uses different materials, and has a slightly different design. However, fitting data in this way introduces the problem of parameter fitting and the freedom that comes with that: since a flexible model can reproduce very different data patterns with different parameter values, changing parameter values from simulation to simulation might lead to good fits not because the model captures reality, but because of its inherent flexibility. That is why we chose to keep parameters at values that were already used in a different publication [5], even if this comes at the cost of perhaps a suboptimal fit of the modeled experimental data.

In the current paper, we simulate several new paradigms. This forced us to introduce new parameters, such as the difference in frequency between ‘high-frequent’ and ‘low-frequent’ items in our lexical disambiguation simulation and the overlap between contexts in the source monitoring simulation (no new ‘architectural’ parameters were added). To ascertain that the model results were not dependent on the values we chose for these new parameters, we repeated our simulations with other plausible values. The results of these simulations are reported here.

### Lexical disambiguation.

Our implementation of lexical disambiguation paradigms introduces three additional parameters. The first is the difference in frequency of occurrence between high-frequent and low-frequent word meanings, modeled through frequency of prelearning. The second and third concern the testing situation. In the simulated study of Salisbury [6], the ambiguous word is presented before the context that disambiguates it. To simulate this, the word form is activated first with a random context, representing the situation at the moment when the ambiguous word is presented. After twenty time steps, (i.e., the model’s activity was updated 20 times), this switches to a context in which the word has been studied before, simulating presentation of the disambiguating sentence context. This introduces two parameters: the

Figure 1: Retrieval of the correct meaning – either dominant or subordinate – of ambiguous words in the lexical disambiguation simulation. Shown are results of the intact (Ctrl) and schizophrenic (Schiz) model, as a function of the difference in frequency (number of prelearning trials) between the dominant and subordinate meaning. The gray bar indicates which simulation is reported in the main text.

Figure 2: Retrieval of the correct meaning – either dominant or subordinate – of ambiguous words in the lexical disambiguation simulation. Shown are results of the intact (Ctrl) and schizophrenic (Schiz) model, as a function of the number of iterations between activation of the word cue combined with a random context, and the activation of the context coherent with the correct word meaning. The two contexts don’t overlap. To the right are shown results with an overlap of 50% between the two contexts; the iteration gap is 20. The gray bar indicates which simulation is reported in the main text.

overlap between the random context and the sentence context (after all some contextual information, such as the room in which the test takes place, might be part of both context representations), and the number of time steps between the word onset and the context switch. We repeated our simulation while varying all three parameters.

Figure 1 shows how high-frequency and low-frequency meanings are retrieved, as a function of the frequency difference between these two categories. As can be seen, the effect of frequency becomes stronger with increasing frequency difference. However, the pattern of results remains the same (as long as this difference is bigger than two): the schizophrenia model is preferentially impaired at retrieving low-frequency meanings.

Figure 2 shows how retrieval is influenced by the moment of context onset, and by the overlap between the first new context and the sentence context. Neither variable had much effect on the pattern of results.

### Context in memory

Our simulation of a basic list-learning, memory experiment was not changed with respect to our previous work [5]. However, we rechecked the effects of two parameters on the results for false alarms and intrusions, reported in the current study. The first parameter concerns the size of the reduction in connections in the schizophrenia model. In addition to the 50% reduction reported in the main document (and in Talamini et al., 2005 [5]), we also ran simulations with reductions of 75% and 25%. Figure 3 shows that both intrusions and false alarms, expressed as proportion of correct recall and recognition respectively (as in the main text), increased approximately linearly with lesion size, with the exception of intrusions at the largest lesion size. With only 25% of connections remaining, both correct free recall and the number of intrusions decreased, but the stronger decrease in intrusions led to a slightly lower intrusion/recall ratio than with the 50% lesion.

The other parameter we investigated was cue size. In the free recall simulations reported in the main text the context cue was set to 75% of the full pattern, while for recognition, a 75% item cue was added. This comes down to 6 of 8 nodes for each pattern. We reran our simulations with cue sizes varying from 5 to 8 nodes (i.e., 62.5% to 100% of the pattern). In recognition the item and context cue were varied together. Figure 4 shows that the size of the cues did not much affect the pattern of results: for all cue sizes, intrusions and false alarms were increased in the schizophrenia model.

### Source monitoring

Our source-monitoring simulation introduced one extra parameter. In the paradigm, items are learned in two overlapping contexts (the two ‘sources’ of information). This overlap was set at 50%, or 4 of 8 nodes in the simulation reported in the main document. Figure 5 reports a simulation in which the overlap between contexts was varied from 25% to 75% (or 2 to 6 nodes of 8). At all overlap levels, the basic result remained the same: decreased source hit levels in the schizophrenia model, and increased proportions of source errors and false alarms.

Figure 3: Intrusions, expressed as a proportion of correct recall, and false alarms (FA), expressed as a proportion of correct recognition (hits), as a function of the reduction in connectivity in the model. The two conditions reported in the main text are indicated by gray bars: the intact model and the 50% reduction schizophrenia model.

Figure 4: Intrusions, expressed as a proportion of correct recall, and false alarms (FA), expressed as a proportion of correct recognition (hits), as a function of the size of item and context cues. The results reported in the main text are indicated by a gray bar.

## Methodological considerations

The model makes several reasonable simplifications with regard to MTL anatomy. Although relinquishing some precision in the mapping of the model onto the real circuitry, these simplifications allowed us to study episodic memory as a process involving sensory integration on the one hand, and storage with orthogonalization on the other hand. The neural network requirements underlying these two processes and their differential contributions to episodic memory could thus be investigated in a more transparent manner than if all (para)hippocampal subdivisions had been considered separately. Here the main simplifications in the model with respect to the biological circuitry are discussed.

Figure 5: Source hits, source errors, and false alarms (source attributions to unstudied words) for the intact and schizophrenia model as a function of the overlap between the two contexts in the source monitoring simulation. The results reported in the main text are indicated by a gray bar.

### Integration of object and context information

In the biological circuitry, integration of information likely occurs over a number of pathways, including several projections from the parahippocampal regions to the hippocampus, as well as horizontal projections between parahippocampal regions. We have abstracted some of these connections away, and assigned the integration process to one connection, namely the feedforward projections from the parahippocampal cortex and perirhinal cortex onto the entorhinal cortex (EC). The notion that an important part of object-place integration occurs in the EC is in line with electrophysiological experiments in rats, showing that in the EC some neurons are responsive to object identity, some are responsive to object position and some are responsive to a combination of the two [7]. Parahippocampal areas at lower levels of the hierarchy appear to be more specifically oriented to either object processing or spatial processing. For instance, during a delayed match to sample task in monkeys nearly half of all visually responsive neurons in LEC are stimulus selective, versus nearly all visually responsive neurons in perirhinal cortex [7,8].

The horizontal connections within the parahippocampal gyrus have different effects depending on the plasticity of the connections. Weight changes during the study phase would help make the model more context sensitive by increasing links between contexts and items. Pre-existing connection strength counteracts this by functioning as noise (i.e., input that is orthogonal to the newly learned associations). The effect of intra-parahippocampal connections would thus depend on the balance between pre-existing weight strength and plasticity. However, little is known about the plasticity of these connections.

### Orthogonalization

Orthogonalization of representations entering the hippocampal circuitry appears to occur in two sequential steps, which in our model are condensed into one step. The first step occurs in the entorhinal to dentate guyrus connection, the second in the dentate gyrus to CA3 connection. Theoretical studies [4] have shown that a double orthogonalization leads to more extensive orthogonalization than with a single step. Thus, the difference with respect to our simplified model is likely to be of a quantitative rather than qualitative nature.

### Feedback projections

While the relative densities of the feedforward connections in the model reflect relative densities in reality, the feedback connections are full (i.e. each node in the source layer connects to each node in the target layer). We chose a full feedback connectivity not because of anatomical considerations, but for technical reasons. Weights and thresholds in the model are computed in a principled fashion from the number of feedforward connections. These values can only be computed when there are only a few sources of variance is limited. A decrease in feedback connectivity would introduce a new source of variance, and would make our calculations impossible. In the case of entorhinal-to-input connections, the feedback is nothing more than a way to read activity in the EC module, since nodes in the input layers do not in fact become active as a result of feedback. These connections are thus themselves not crucial to the model. .

## References

## 1. Oja E (1982) A simplified neuron model as a principal component analyzer. J Math Biol 15: 267-273.

## 2. Levy WB, Colbert CM, Desmond NL (1990) Elemental adaptive processes in neurons and synapses: A statistical/computational perspective. In: Gluck MA, Rumelhart DE, editors. Neuroscience and connectionist theory. Hillsdale, NJ: Lawrence Erlbaum.

## 3. Hebb DO (1949) The organization of behavior. New York: Wiley.

## 4. O'Reilly RC, McClelland JL (1994) Hippocampal conjunctive encoding, storage, and recall: Avoiding a trade-off. Hippocampus 4: 661-682.

## 5. Talamini LM, Meeter M, Murre JMJ, Elvevåg B, Goldberg TE (2005) Reduced parahippocampal connectivity produces schizophrenia-like memory deficits in simulated neural circuits. Arch Gen Psychiatry 62: 485-493.

## 6. Salisbury DF, Shenton ME, Nestor PG, McCarley RW (2002) Semantic bias, homograph comprehension, and event-related potentials in schizophrenia. Clin Neurophysiol 113: 383-395.

## 7. Suzuki WA, Miller EK, Desimone R (1997) Object and place memory in the macaque entorhinal cortex. J Neurophysiol 78: 1062-1081.

## 8. Miller EK, Li L, Desimone R (1993) Activity of neurons in anterior inferior temporal cortex during a short-term memory task. J Neurosci 13: 1460-1478.
